# American Public Health Association

**Published:** 1881-10-20

**Authors:** 


### *AMERICAN PUBLIC HEALTH ASSOCIATION.*

This Association is appointed to meet at Savannah, November 29th to December 2d, 1881. We trust that the profession everywhere, and especially in our own State, will take an interest in the Association. Certainly no more important object can be found than the preservation of the public health. It lies at the foundation of all that is noble in medical science. The physician is as truly the conservator of the public health as the officers of our country are the preservers of the peace and safety of the public. Every true physician will rise above the mere pecuniary consideration which attaches to his profession, and realize the moral responsibility which his superior knowledge in respect to disease and sanitary science gives him over all others in the community. The time is coming, and now is, when the public will look to us for the solution of scientific problems of great and inestimable importance connected with the public health. That we have so long failed to discover the hidden causes of those appalling and destructive epidemics which almost annually sweep over certain sections of our country, must soon become, if it has not already done so, an opprobrium to the boasted advances in medical science. Let, then, the profession come together and consult on every possible occasion, and persevere in their sanitary efforts until light and truth shall reward their labors.

